# Case Report: A Preferred Reconstructing Modality to Restore Neoplastic Nasal Alar Subunit Defects: Sequential Facial Artery Perforator Flaps

**DOI:** 10.3389/fsurg.2021.796990

**Published:** 2022-01-20

**Authors:** Bihua Wu, Sanhong Yang, Hai Li, Tianhua Zhang, Shune Xiao, Zairong Wei, Chengliang Deng

**Affiliations:** Department of Plastic Surgery, Affiliated Hospital of Zunyi Medical University, Zunyi, China

**Keywords:** facial artery perforator flap, nasal alar, neoplastic nasal alar defects, nasolabial groove, nasal alar subunit

## Abstract

**Background:**

Achieving perfect repair of a nasal defect with the recovery of cosmetic subunits has become a challenge to plastic, dermatologic, and head and neck surgeons. The study aimed to evaluate the effect of reconstructing neoplastic nasal alar subunit defects with sequential facial artery perforator flaps produced from nasolabial groove tissue.

**Methods:**

A retrospective analysis of 20 patients who had undergone reconstruction for neoplastic nasal alar defects with this technique from January 2017 to October 2019 was performed. The reconstruction procedure used sequential facial artery perforator flaps. The surgical procedure used and follow-up results achieved have been documented photographically for all patients.

**Results:**

The aesthetic and functional results of surgery were satisfactory in all the 20 patients. After all surgeries, the reconstructed alar tissues were compliant, bilateral symmetries of the alae and nasolabial grooves were satisfactory, and no patients exhibited color mismatches between the flaps and surrounding tissues. During a mean follow-up period of 22 months, none of the patients exhibited alar retraction, inferior displacement, deformation, or hypertrophic scarring.

**Conclusions:**

The sequential facial artery perforator flap technique created with nasolabial groove tissue to reconstruct neoplastic nasal alar defects is a simple single-stage procedure that provides excellent surgical outcomes.

## Introduction

The incidence of malignant facial skin tumors is increasing, and the nose and adjacent regions are highly vulnerable sites for skin tumor development (1). Approximately 33% of nasal skin tumors affect the alar area (2). After alar tumor resection, the remaining defect usually involves multiple cosmetic units, destroying the supra-alar crease, alar-facial groove, and the melolabial fold (1). Achieving a perfect nasal defect repair with the recovery of cosmetic units has become a challenge for plastic surgeons, dermatologic surgeons and head and neck surgeons. Direct closure and full-thickness skin grafts are common therapeutic options for alar defect reconstruction (3). However, direct closure may easily cause alar rim distortion and is only suitable for small defects. Skin atrophy and hyperpigmentation often occur in the later stages of skin graft healing and affect the aesthetic appearance of the grafts. Moreover, using a graft from a local skin flap is the preferred approach for alar defect reconstruction because of its improved cosmetic outcomes reported (4, 5). The texture and color of the local flap of the nose are very similar to those of the defect area. However, distortion and asymmetry often appear after reconstruction with a nasal local flap, especially in large-area defects, and are attributed to the lack of reservoir tissue in the nose. Therefore, other regions of the skin adjacent to the nose, such as the skin from the nasolabial groove and cheek, are frequently used to create flaps (6) and are particularly favorable for defects affecting more than 50% of the nasal aesthetic subunits (7). Among the flap procedures, the facial artery perforator flap method is good for repairing defects of the alar subunit (8–10). This study aimed to describe the use of the sequential facial artery perforator flap created from nasolabial fold tissues to reconstruct neoplastic nasal alar subunit defects with the achievement of an appropriate cosmetic result.

## Methods

### Patients

Twenty patients with basal cell carcinoma in alar area, diagnosed pre-operatively by biopsy, were enrolled at the Department of Plastic Surgery, Affiliated Hospital of Zunyi Medical University between January 2017 and October 2019. There were 14 were male and six female patients, with a mean age of 64 years (range: 50–83 years). Inclusion criteria included (1) having a primary tumor in the alar area with pre-operative biopsy showing basal cell carcinoma, (2) frozen sections showing that the alar cartilage was not affected by tumor, (3) the defect sizes ranged from 1.5 × 1.5 cm to 2.5 × 2.5 and (4) being 18-85 years of age. Exclusion criteria included: (1) history of surgery in the region of facial artery distribution on the side of the tumor, (2) history of receiving local radiation therapy for the tumor, (3) current serious hematologic, immunologic, or circulatory disease, and any contraindications to surgery.

### Surgical Procedures

**Figure 1:**
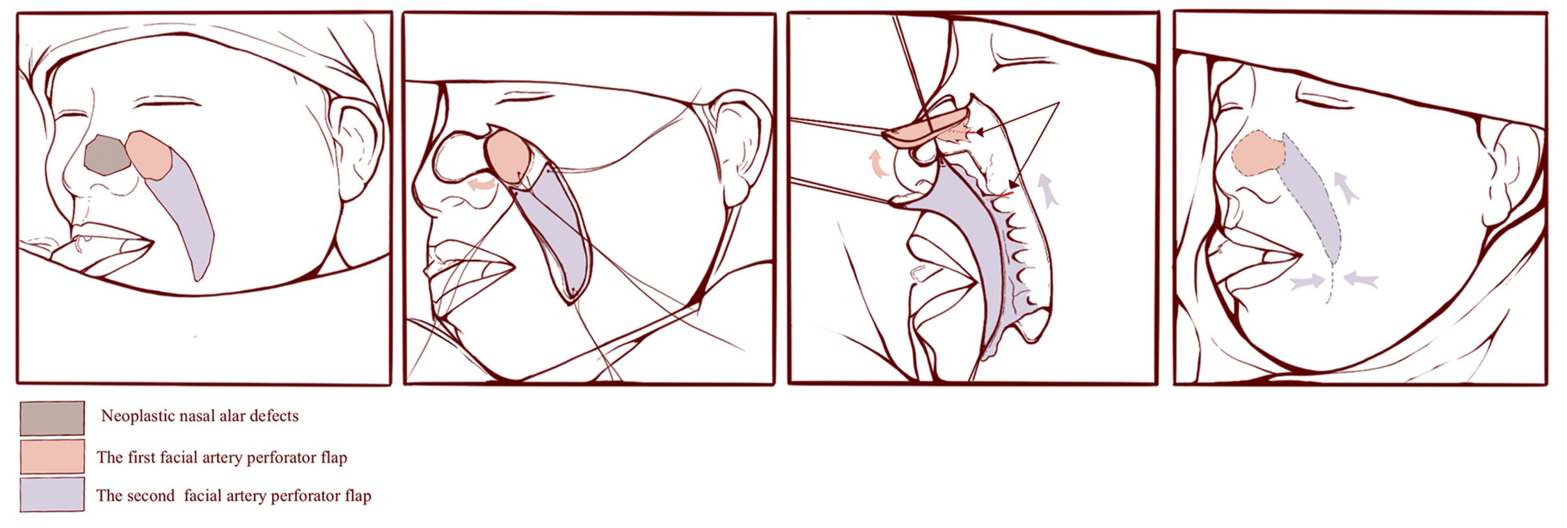
Schematic illustration of the surgical procedures.

Tumor resections and defect reconstruction procedures were performed under general anesthesia. The tumor was resected with a 0.5-cm margin, down to the surface of alar cartilage. Rapid intraoperative histopathological examinations were performed on the frozen sections obtained. Reconstructive surgery was conducted following confirmation from histopathology results revealing complete tumor ablation. Considering that the facial artery distributes many branches to supply the skin of nose and face, we used the perforating branch of the lateral nasal artery, arising from the facial artery, to design a round flap to repair the nasal alar defect, and the perforator of the major branch of the facial artery in the nasolabial groove areas to design the V-Y flap for repair of the donor site for the round flap. Doppler was used to locate and mark the course of the facial artery, and flaps were designed based on the anatomical information of the facial artery and its tributaries. Initially, the first, round facial artery perforator flap, of same size as the defect, was elevated and rotated clockwise to repair the defect, and the second V-Y facial artery perforator flap was elevated and advanced to repair the donor site for the first flap. The flaps were elevated in the superficial fascia plane under a head-mounted microscope, 5–0 absorbable sutures were used to suture the dermis and 6–0 nylon sutures were used to suture the skin (Figure 1).

### Postoperative Details

Patients were discharged 2–3 days postoperatively. Examinations and removal of the sutures were performed 1 week postoperatively. The second patient examinations were conducted in the third week after surgery, when silicone ointment was topically administered to treat the scars. Additional patient follow-up examinations occurred every 3 months, the postoperative appearance was observed and oncological surveillance was monitored by dermatoscopy. All patients were followed up for 12–40 months, with an average of 22 months.

### Ethics Statement

The study was approved by the Clinical Research Ethics Committee of the Affiliated Hospital of Zunyi Medical University. All procedures performed in the study involving human participants were in accordance with the Declaration of Helsinki (as revised in 2013). All patients provided preoperative informed consent and granted the authors use of their photographs for scientific purposes.

## Results

The histopathology of postoperative paraffin sections of all patients was basal cell carcinoma and the margins and bases of the wounds were free of tumor cells. The extents of the defects after tumor excision ranged from 1.5 × 1.5 cm to 2.0 × 2.5 cm (Table 1). The nasal cartilage was preserved in all patients. In all the 20 patients, the color of the flap was ruddy, and the capillary response was normal. No partial or total necrosis, infection, or venous insufficiency was observed in any of the flaps, and no patient required a second surgery. Scars in the operation areas were not noticeable 12 months after the surgeries.

**Table 1:** Characteristics of patients.

**No**	**Age/sex**	**Primary T**	**Involved aesthetic subunits**	**Defect size**	**Margins status in the finally report**	**Follow-up**	**Recurrence**
1	68/Male	Basal cell carcinoma (BCC)	Right alar	1.5 × 1.8 cm	Free of tumor	12	No
2	59/Female	BCC	Left alar	1.8 × 2.0 cm	Free of tumor	15	No
3	52/Male	BCC	Left alar	1.5 × 1.5 cm	Free of tumor	30	No
4	61/Male	BCC	Right alar	2.0 × 2.5 cm	Free of tumor	33	No
5	51/Male	BCC	Right alar	1.9 × 2.2 cm	Free of tumor	13	No
6	70/Male	BCC	Right alar	2.0 × 1.5 cm	Free of tumor	17	No
7	77/Female	BCC	Right alar	1.6 × 1.8 cm	Free of tumor	20	No
8	58/Male	BCC	Left alar	1.7 × 2.0 cm	Free of tumor	18	No
9	62/Male	BCC	Right alar	1.8 × 2.0 cm	Free of tumor	22	No
10	50/Female	BCC	Right alar	1.5 × 1.5 cm	Free of tumor	31	No
11	69/Female	BCC	Right alar	2.0 × 2.2 cm	Free of tumor	40	No
12	83/Female	BCC	Left alar	1.6 × 1.8 cm	Free of tumor	31	No
13	60/Female	BCC	Left alar	1.8 × 2.1 cm	Free of tumor	28	No
14	65/Female	BCC	Left alar	1.6 × 1.6 cm	Free of tumor	18	No
15	62/Male	BCC	Left alar	1.7 × 1.9 cm	Free of tumor	27	No
16	84/Male	BCC	Right alar	1.6 × 1.8 cm	Free of tumor	15	No
17	69/Male	BCC	Left alar	2.0 × 2.5 cm	Free of tumor	25	No
18	71/Female	BCC	Right alar	1.7 × 1.9 cm	Free of tumor	17	No
19	62/Male	BCC	Right alar	1.8 × 1.8 cm	Free of tumor	13	No
20	51/Male	BCC	Right alar	1.7 × 1.5 cm	Free of tumor	13	No

**Figure 2:**
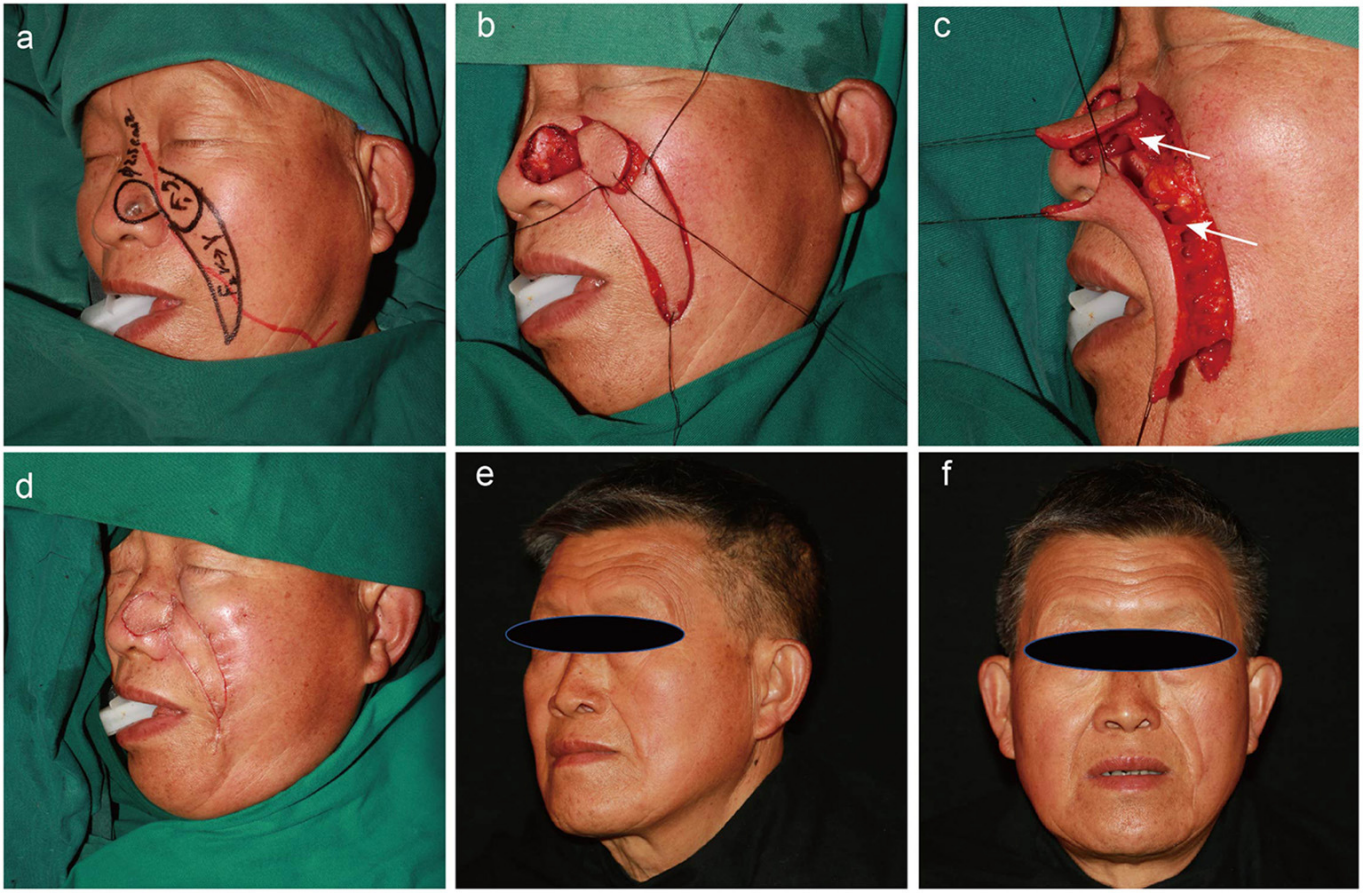
A 69-year-old man with a history of basal cell carcinoma on the left ala for 2 years. Different steps of the operation and postoperative follow-up (25 months). **(a)** Preoperative design. The red line shows the location of the facial artery. **(b,c)** Dissection of the round rotational flap and V–Y advancement island pedicle flap. The white arrow indicates the perforating branch of the facial artery. **(d)** Flaps are sutured into the defect using a dermal suture with 5–0 absorbable sutures. **(e,f)** Appearance at 25 months after the operation.

**Figure 3:**
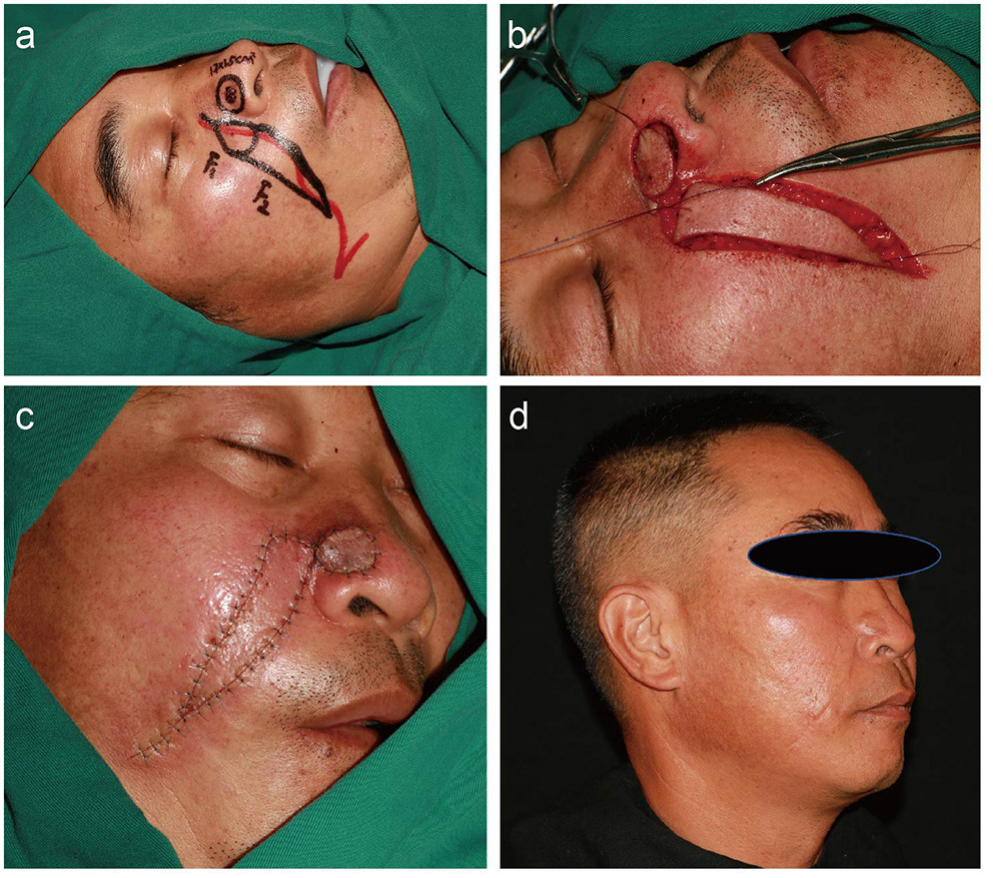
A 51-year-old man with a history of basal cell carcinoma on the right ala for 7 months. **(a)** Preoperative design. The red line shows the location of the facial artery. **(b)** Dissection of the round rotational flap and V–Y advancement island pedicle flap. **(c)** Flaps are sutured into the defect using dermal suture with 5–0 absorbable sutures and a skin suture with 6-0 nylon suture. **(d)** Appearance at 13 months after the operation.

**Figure 4:**
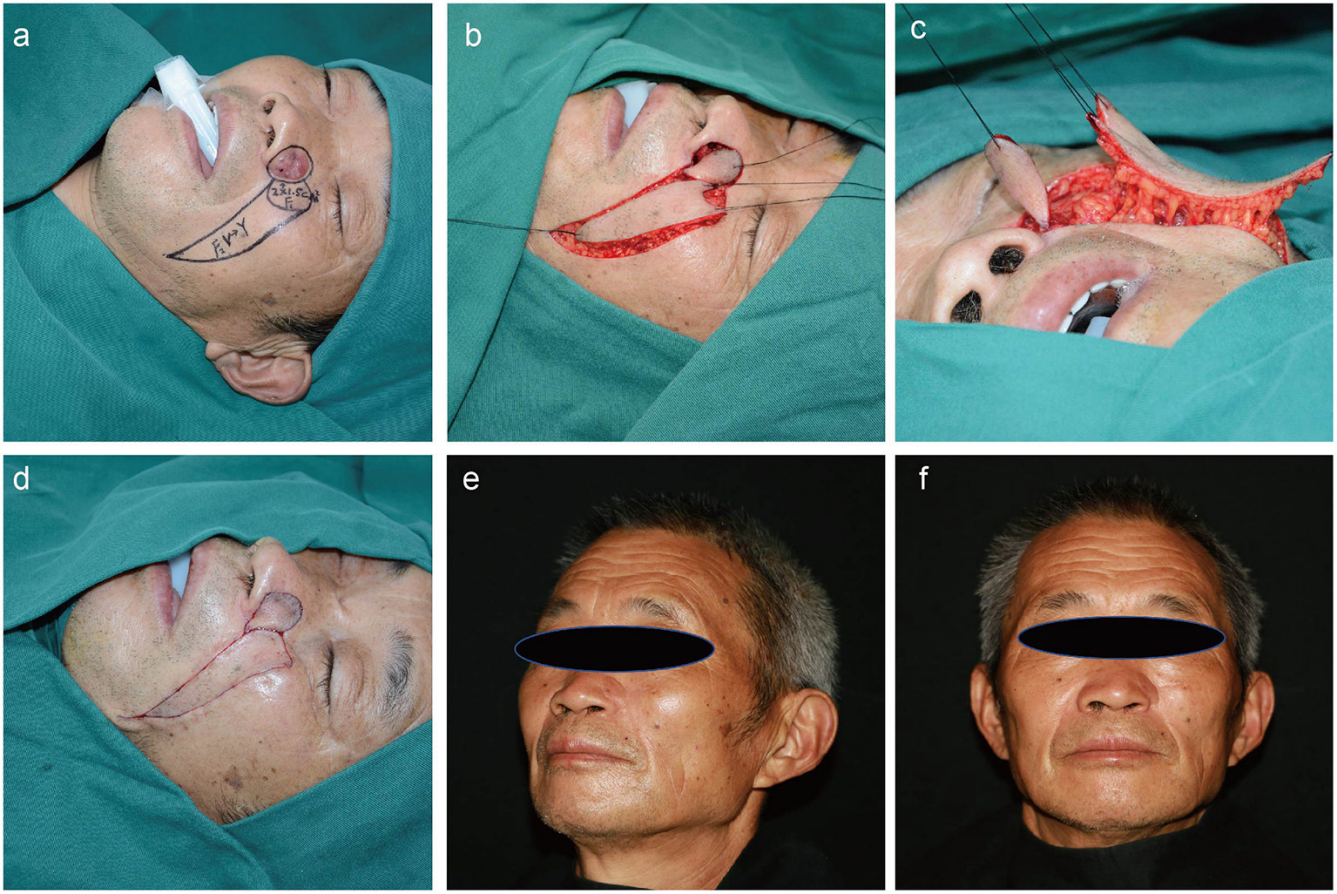
A 70-year-old man with a history of basal cell carcinoma on the left ala for 1 year. Application of the sequential flap in a third patient. **(a)** Preoperative design. **(b,c)** Dissection of the round rotational flap and V–Y advancement island pedicle flap. **(d)** Flaps are sutured into the defect using a dermal suture with 5–0 absorbable sutures. **(e,f)** Appearance at 17 months after the operation.

All flaps integrated well with the surrounding tissues. The reconstructed alar tissues were compliant and bilateral symmetries of the alae and nasolabial grooves were satisfactory for each patient. None of the patients exhibited color mismatches between the flaps and surrounding tissues. During a mean post-surgery follow-up time of 22 months, no patients showed tumor recurrence, and none of the patients exhibited retraction, inferior displacement, deformation, or scarring of the alar tissues. Thus, the aesthetic and functional results of all patients were satisfactory (Figures 2–4).

### Case Reports

#### Patient 1

A 69-year-old man was admitted to our department with a 2-year history of a malignant skin tumor on the left ala. A pre-operative biopsy showed basal cell carcinoma. The defect after tumor enlarged excision was 2.0 × 2.5 cm. A flap surgery was performed using the sequential facial artery perforator flaps method; afterwards, tumor ablation and negative frozen section were commenced.

The flaps survived and the wounds healed by primary intention. During a 25-month patient follow-up period, the tumor did not relapse, and the flap maintained an excellent appearance and texture. In addition, the alar tissue was compliant and bilateral symmetries of the alae and nasolabial grooves were satisfactory (Figure 2).

#### Patient 2

A 51-year-old man with a 7-month history of a malignant skin tumor on the right ala was referred to our department. A pre-operative biopsy showed basal cell carcinoma. Rapid intraoperative histopathological examination of frozen sections confirmed complete tumor ablation. Excision of the tumor produced a skin defect that was 1.7 × 1.5 cm and the sequential facial artery perforator flaps method was chosen for wound repair. The flaps survived and the wounds healed by primary intention. The patient was followed for 13 months, with no tumor recurrence, and the surgical results were satisfactory (Figure 3).

#### Patient 3

A 70-year-old man was referred to our department with a 1-year history of a malignant skin tumor on the right ala. A pre-operative biopsy examination revealed that the tumor was a basal cell carcinoma. Following tumor resection and negative margins on frozen section, the 2.0 × 1.5-cm skin defect was repaired with sequential facial artery perforator flaps. The flaps survived and the wounds healed by primary intention. The patient was followed for 17 months, with no tumor recurrence, and satisfactory results were achieved (Figure 4).

## Discussion

Basal cell carcinomas (BCCs) are common skin cancers that often affect the nose and adjacent regions. Surgical excision and Mohs surgery are the most commonly used because of their association with a low recurrence rate and the ability to confirm residual tumor pathologically (11). The nose is one of the most important cosmetic units of the face. The nasal cosmetic subunits have been identified as the dorsum, tip, alar, columella, soft-tissue triangles, and paired sidewalls (12). The best cosmetic outcome of an alar defect repair results from reconstruction of the defect within the subunit. Preserving the natural alar contour and contralateral symmetry in alar reconstruction is important (13). However, the structure of the alar area is rigid, the mobility is low, and the effects of some traditional reconstruction techniques, such as direct closure and skin grafting, are unsatisfactory (3). The use of local flaps to reconstruct the nasal alae has achieved good aesthetic results (14, 15). However, due to the lack of reservoir tissue in the nose, many limitations for reconstructive surgery still exist. In addition, some other alar repair methods have shown satisfactory results; however, those procedures are complicated (16, 17).

Previous studies have reported that the facial arterial perforator flap provides satisfactory results for repair of alar defects (8, 9). However, the flap pedicle used in this procedure required tissue removal and additional treatment. In addition, the alar groove can become shallower and less attractive with this method. In our study, sequential facial artery perforator flaps were used to reconstruct the neoplastic nasal alar subunit defects. The first flap was used to repair the neoplastic skin defect and reconstruct the lateral alar sulcus and the second flap was used to repair the donor area for the first flap, thereby avoiding the additional treatment of pedicle tissue and reducing deformation. This was a one-step intervention that fulfilled all the above requirements with minimal complications. Additionally, the procedure was simple and easy to master. In the previous study, the three-dimensional architecture of the facial artery's distribution reckoned from high-resolution angiograms was reported, which provided key information for the safe manipulation of reconstructive flaps during surgery (18). Nasolabial groove tissue was used as reservoir tissue, which is very appropriate in the nose repairing; the perforators of the facial artery were used as the vascular pedicles, which provided a reliable blood supply for the flaps.

Nasal alar defect after oncologic resection is common in clinical practice. In our study, the reconstruction of defects with an extent of up to 2.0 × 2.5 cm showed excellent surgical outcomes using relay facial artery perforator flaps. However, the limitation of this technique is that it may only be effective for defects in the alar subunits that are no more than 2.5 × 2.5 cm and without alar cartilage defects. Thus, a paramedian forehead flap should be considered if the defect involves multiple cosmetic subunits of the nose and a cartilage graft should be combined for full-thickness nasal alar defects. In addition, incisions from sequential flaps have an increased risk of scarring, especially in East Asian patients. Therefore, postoperative anti-scar treatment is very important. Moreover, patients with a history of surgery in the facial artery distribution area ipsilateral to the tumor and patients who have underwent preoperative radiotherapy are not suitable for this method of repair because the facial artery perforators are likely to have been damaged. In our study, all the patients that were followed were satisfied with their surgical results. The sequential facial artery perforator flaps method using nasolabial groove tissue is a simple, single-stage procedure for the reconstruction of neoplastic nasal alar defects that leads to excellent surgical outcomes.

## Data Availability Statement

The original contributions presented in the study are included in the article/supplementary material, further inquiries can be directed to the corresponding author.

## Ethics Statement

Written informed consent was obtained from the individual(s) for the publication of any potentially identifiable images or data included in this article.

## Author Contributions

BW and CD contributed to the conception and design and manuscript writing. SY and SX contributed to the data collection. TZ and HL contributed to the follow-up of patients. ZW, BW, and CD contributed to the surgery. All the authors read and approved the final manuscript.

## Funding

This work was funded by the National Natural Science Foundation of China (no. 81801921), Science and Technology Fund Project of Guizhou Provincial Health Commission (gzwjkj2020-1-115), Science and Technology Program of ZunYi (2019-49, 2019-51), the PhD Fund of Scientific Research Foundation of ZunYi Medical University (2018-10, 2018-14), and Fund of Affiliated Hospital of Zunyi Medical University (2014-29).

## Conflict of Interest

The authors declare that the research was conducted in the absence of any commercial or financial relationships that could be construed as a potential conflict of interest.

## Publisher's Note

All claims expressed in this article are solely those of the authors and do not necessarily represent those of their affiliated organizations, or those of the publisher, the editors and the reviewers. Any product that may be evaluated in this article, or claim that may be made by its manufacturer, is not guaranteed or endorsed by the publisher.
